# Risk factors for portal hypertensive gastropathy

**DOI:** 10.1186/s12876-022-02468-7

**Published:** 2022-10-14

**Authors:** Ran Wu, Kunyi Liu, Chengyi Shi, Hui Tian, Na Wang

**Affiliations:** Department of Gastroenterology, The Second Hospital of Hebei Medical University, Hebei Key Laboratory of Gastroenterology, Hebei Institute of Gastroenterology, Hebei Clinical Research Center for Digestive Diseases, 215 Heping West Road, Shijiazhuang, 050000 Hebei Province China

**Keywords:** Cirrhosis, Portal hypertensive gastropathy, Oesophagogastric varices, Liver function

## Abstract

**Background:**

Portal hypertensive gastropathy (PHG) is often underestimated in clinical diagnosis. Gastrointestinal bleeding in cirrhosis of PHG accounts for approximately 10% of upper gastrointestinal bleeding. However, the relationship between PHG and gender, laboratory parameters, liver function and varices is still controversial. In the present study, we aimed to retrospectively evaluate the incidence of PHG and to explore the relationship between PHG and gender, laboratory parameters, liver function and varicose veins.

**Methods:**

A retrospective analysis of 325 patients with cirrhosis who underwent esophagogastroduodenoscopy (EGD) in the Department of Gastroenterology of the Second Hospital of Hebei Medical University from 1 January 2018 to 31 December 2020 was performed. The relationships among age, gender, laboratory parameters, Child–Pugh stage, oesophageal varices (EV), gastric varices (GV) and ascites with PHG were analysed with univariate and multivariate logistic regression.

**Results:**

The occurrence of PHG was significantly associated with gender, haemoglobin, platelet count, prothrombin time, albumin, Child–Pugh stage, EV, GV and ascites (*P* < 0.05). Furthermore, there was a positive correlation between the severity of PHG and the degree of EV, GV and ascites (*P* < 0.05). Multivariate logistic regression showed that albumin, EV and GV levels were independently associated with the occurrence of PHG.

**Conclusion:**

The incidence of PHG in cirrhosis was 87.4% in this study. The occurrence of PHG was related to gender, haemoglobin, platelet count, prothrombin time, albumin, Child–Pugh stage, EV, GV and ascites. Albumin, the degree of EV and GV are independent risk factors for the occurrence of PHG.

## Introduction

Portal hypertensive gastropathy (PHG) is a common complication of liver cirrhosis and is often underestimated in clinical diagnosis [1]. The incidence of PHG is approximately 20% to 98% in patients with cirrhosis [2, 3]. Gastrointestinal bleeding in patients with cirrhosis of PHG accounts for approximately 10% of upper gastrointestinal bleeding and is second only to oesophageal variceal bleeding in the cause of upper gastrointestinal bleeding [4]. Severe PHG can cause severe anaemia and can even be life-threatening. However, the relationship between PHG and gender, laboratory parameters, liver function and varices is still controversial. Therefore, more attention should be given to PHG, and risk factors for the occurrence and development of PHG should be identified.

Most studies found no association between PHG and the etiology of cirrhosis or gender. However, Simbrunner et al. [5] pointed out that the male ratio of patients with severe PHG was significantly higher than that of patients with mild PHG and patients without PHG. At present, the relationship between the occurrence of PHG and age, gender, etiology, bilirubin, albumin and other laboratory indicators has not reached a consensus.

The relationship between PHG and varices is still inconsistent. Marrache et al. [6] concluded that there was a weak correlation between hepatic venous pressure gradient (HVPG) levels and the presence of PHG. Kumar et al. [7] found a significant correlation between the size of oesophageal varices (EV) and the presence of PHG. However, Saleem et al. [8] and Tiwari et al. [2] found the opposite and suggested that the grade of EV is not related to the severity of PHG.

Meanwhile, the relationship between PHG and Child–Pugh stage of liver function is still inconsistent. Mandhwani et al. [9], Sathar et al. [10] and Tiwari et al. [2] showed that there was a significant association between the occurrence of PHG and Child–Pugh stage. In contrast, El-Kalla et al. [11] found no difference between them with respect to Child–Pugh stage. The major purpose of this study was to retrospectively evaluate the incidence of PHG and explore the relationship between PHG and gender, laboratory parameters, liver function and varicose veins.

## Materials and methods

### Recruitment of participants

A retrospective analysis of patients with cirrhosis who underwent esophagogastroduodenoscopy (EGD) in the Department of Gastroenterology of the Second Hospital of Hebei Medical University from 1 January 2018 to 31 December 2020 was performed. The diagnosis of cirrhosis was based on history, clinical examination, laboratory parameters, imaging diagnosis and/or histopathological examination. Patients with cirrhosis with complete data were included and not limited to the etiology of cirrhosis. Patients who used antibacterial drugs, antithrombotic drugs, nonsteroidal anti-inflammatory drugs, acid suppressor or gastric mucosal protection drugs in the past month or who underwent TIPS, surgical shunt, flow interruption or liver transplantation were excluded. Patients with active upper gastrointestinal bleeding or with ongoing comorbid conditions, such as acute exacerbation of chronic obstructive pulmonary disease/asthma and myocardial infarction (within six months), and patients on the ventilator were excluded. Patients with hepatocellular carcinoma, portal vein or splenic vein thrombosis, acute-on-chronic liver failure or noncirrhotic portal hypertension were excluded. The patient's gender, age, etiology, white blood cell count, haemoglobin, platelet count, total bilirubin, direct bilirubin, indirect bilirubin, alanine aminotransferase, aspartate aminotransferase, prothrombin time and creatinine were recorded. The severity of ascites, oesophagogastric varices and PHG were recorded.

### Criterion

The severity of liver disease was stratified based on Child–Pugh stage. The severity of ascites was graded according to the European Association for the Study of the Liver: small: detectable only by ultrasound; medium: moderately symmetrical abdominal distension; abounding: abounding ascites with marked abdominal distention. The severity of EV and GV were graded according to the American Association for the Study of Liver Diseases [12]. The severity of PHG was graded according to the Baveno scoring system (Table 1). Three endoscopists with more than ten years of experience independently evaluated all the endoscopy images. They were all blinded to the clinical data of the patients. Cases of disagreement were discussed and resolved by consensus, according to the Baveno scoring system.

**Table 1:** Baveno scoring system

Endoscopic features	0	1	2
Mucosal mosaic pattern	No	Mild	Severe
Red spots	No	Isolated	Confluent
Gave	No	**–**	Existent

Scores = 0: no; scores ≤ 3: mild; scores ≥ 4: severe

### Statistical analysis

All statistical analyses were executed by using SPSS 25 software. Continuous variables are expressed as the mean (± SD), and discrete variables are expressed as numbers and percentages. Continuous variables were compared using Student’s t test or the Mann–Whitney test as appropriate, and discrete variables were compared using the chi-square test or Fischer’s exact test as appropriate. The Spearman correlation coefficient was used to assess bivariate correlation. A two-sided *P* value of < 0.05 was considered significant.

### Ethical review

This study was conducted according to the principles expressed in the Declaration of Helsinki and approved by the Scientific Research Ethical Licensing Committee of the Second Hospital of Hebei Medical University. Due to the retrospective study design, written informed consent was waived (Scientific Research Ethical Licensing Committee of the Second Hospital of Hebei Medical University).

## Results

A retrospective analysis of 325 patients with cirrhosis hospitalized in the Department of Gastroenterology of the Second Hospital of Hebei Medical University was performed. There were 284 patients diagnosed with PHG. The incidence of PHG in cirrhosis was 87.4%, including 247 cases (76.0%) with mild PHG and 37 cases (11.4%) with severe PHG. There were no significant differences regarding age, etiology, white blood cells (WBC), alanine aminotransferase (ALT), aspartate aminotransferase (AST), total bilirubin (TBIL), direct bilirubin (DBIL), indirect bilirubin (IBIL) or creatinine (Cr) between the PHG group and the non-PHG group. In contrast, there were 187 males (65.8%) and 97 females (34.2%) in the PHG group and 20 males (48.8%) and 21 females (51.2%) in the non-PHG group. The proportion of males in the PHG group was higher than that in the non-PHG group (*P* < 0.05). There were significant differences in gender, haemoglobin (Hb), platelet count (PLT), prothrombin time (PT) and albumin (ALB) between the two groups (*P* < 0.05). (Table 2).

**Table 2:** Comparison of data between the two groups

Project	Non-PHG	PHG	Statistic	*P*
Age	57.00 (46.00,63.00)	54.00 (46.75,60.00)	− 1.117	0.264
Gender			4.512	0.034
Male	20	187		
Female	21	97		
Etiology			2.979	0.519
Hepatitis B	23	155		
Hepatitis C	5	20		
Alcohol	1	7		
Cholestasis	2	9		
Other	10	93		
WBC (× 10^9^/L)	4.10 (2.91–5.71)	3.60 (2.56–4.60)	− 1.856	0.063
Hb (g/L)	111.00 (95.00–139.50)	106.00 (80.00–122.00)	− 2.595	0.009
PLT (× 10^9^/L)	90.00 (70.00–138.50)	40.00 (47.75–97.25)	− 3.859	0.000
PT (s)	13.60 (11.90–15.15)	14.40 (13.10–16.50)	− 2.526	0.012
TBIL (umol/L)	21.00 (14.05–39.30)	20.50 (13.77–35.47)	− 0.067	0.947
DBIL (umol/L)	9.50 (6.30–19.15)	10.35 (6.90–19.92)	− 0.454	0.650
IBIL (umol/L)	9.70 (7.02–13.15)	8.85 (6.27–14.30)	− 0.610	0.542
ALB (g/L)	35.79 ± 6.31	32.92 ± 6.14	2.732	0.009
ALT (U/L)	30.70 (17.75–46.40)	29.75 (18.45–45.02)	− 0.402	0.688
AST (U/L)	30.50 (23.70–53.75)	39.00 (26.07–60.40)	− 1.695	0.090
Cr (umol/L)	65.00 (51.00–73.50)	64.00 (55.00–76.00)	− 0.604	0.546

There were 154 cases of Child–Pugh A patients, including 27 cases without PHG, 108 cases with mild PHG and 19 cases with severe PHG. There were 121 Child–Pugh B patients, including 12 without PHG, 97 with mild PHG and 12 with severe PHG. There were 50 cases of Child–Pugh C grade patients, including 2 cases without PHG, 42 cases with mild PHG and six cases with severe PHG. There was a statistically significant difference between Child–Pugh class and the incidence of PHG (H = 8.003, *P* = 0.018). However, there was no linear relationship between Child–Pugh class and the severity of PHG (Spearman correlation coefficient: 0.093, *P* = 0.094) (Table 3).

**Table 3:** Association between the severity of PHG and Child–Pugh stage, EV, GV and ascites

Parameter	PHG			Statistic	*P*
	No	Mild	Severe		
Child–Pugh				8.003	0.018
A	27	108	19		
B	12	97	12		
C	2	42	6		
Ascites					
No	34	138	21	12.152	0.002
Mild	6	55	8		
Moderate	1	47	6		
Severe	0	7	2		
EV					
No	21	18	1	58.139	0.000
Mild	7	40	2		
Moderate	8	54	3		
Severe	5	135	31		
GV					
No	36	110	8	38.525	0.000
Mild	2	8	1		
Moderate	1	73	11		
Severe	2	56	17		

There were 193 patients without ascites, 69 patients with mild ascites, 54 patients with moderate ascites and 9 patients with severe ascites. There was a statistically significant difference between ascites and the incidence of PHG (H = 12.152, *P* = 0.002). Moreover, there was a positive correlation between the degree of ascites and the severity of PHG (Spearman correlation coefficient was 0.151, *P* = 0.007) (Table 3).

Among the 325 cirrhosis cases, there were 40 cases of no EV, including 21 cases without PHG, 18 cases with mild PHG and 1 case with severe PHG. There were 49 cases of mild EV, including 7 cases without PHG, 40 cases with mild PHG, and 2 cases with severe PHG. There were 65 cases of moderate EV, including 8 cases without PHG, 54 cases with mild PHG and 3 cases with severe PHG. There were 171 cases of severe EV, including 5 cases without PHG, 135 cases with mild PHG and 31 cases with severe PHG. There was a statistically significant difference between the degree of EV and the incidence of PHG (H = 58.139, *P* < 0.001). Moreover, there was a positive correlation between the degree of EV and the severity of PHG (Spearman correlation coefficient: 0.419, *P* < 0.001) (Table 3).

Among 325 patients with cirrhosis, there were 154 cases of no GV, 11 cases of mild GV, 85 cases of moderate GV and 75 cases of severe GV. There was a statistically significant difference between the degree of GV and the incidence of PHG (H = 38.524, *P* < 0.001. Moreover there was a positive correlation between the degree of GV and the severity of PHG (Spearman correlation coefficient: 0.343, *P* < 0.001) (Table 3).

In conclusion, there were statistically significant differences in gender, Hb, PLT, PT, ALB, Child–Pugh stage, EV, GV and ascites between the two groups by univariate analysis. Multiple regression analysis found that albumin and the degree of EV and GV significantly affected the incidence of PHG (Table 4).

**Table 4:** Logistic multivariate analysis of risk factors for PHG

	B	SE	Wald	*P*	OR	95% CI
Gender (male)	0.458	0.438	1.093	0.296	1.581	0.670–3.730
Hb	0.011	0.010	1.353	0.245	1.011	0.992–1.030
PLT	-0.008	0.005	2.556	0.110	0.992	0.983–1.002
ALB	-0.120	0.048	6.336	0.012	0.887	0.807–0.974
PT	-0.239	0.130	3.403	0.065	0787	0.611–1.015
Degree of EV	0.908	0.224	16.357	0.000	2.478	1.596–3.847
Degree of GV	0.752	0.278	7.320	0.007	2.120	1.230–3.655
Degree of ascites	0.542	0.438	1.531	0.216	1.719	0.729–4.052
Child–Pugh	0.734	0.578	1.610	0.204	2.083	0.670–6.474

## Discussion

In 1984, Sarfeh et al. [13] proposed that portal hypertension would lead to specific gastric mucosal haemodynamics and morphological changes, manifesting as gastric mucosal hyperaemia, oedema, erosion and even haemorrhage under endoscopy. Therefore, "portal hypertensive gastritis" was proposed. In 1985, McCormack's team [14] described mucosal hyperaemia and mild inflammation in the fundus and body of the stomach. Histological biopsy showed that capillary dilation in the mucosal layer far exceeded mucosal inflammation, and the concept of "congestive gastropathy" was first proposed. In 1986, Sarfeh et al. formally proposed the term "portal hypertensive gastropathy", which is still used today. Endoscopic presentation of gastric vascular ectasia (GAVE) is a flat red spot that is located in the distal stomach (gastric antrum) and often arises in strips from the gastric antrum. It resembles a watermelon, the so-called "watermelon stomach". When it is difficult to distinguish from PHG, biopsy of gastric mucosal lesions should be considered for diagnosis. Histological manifestations of GAVE were telangiectasia, proliferation of lamina propria spindle cells, fibrin thrombus and fibrin deficiency [15].

PHG is a term of endoscopic diagnosis, in which endoscopic features include a typical snakeskin mosaic pattern, flat or bulging red marks or red spots resembling vascular ectasias. The histologic findings of PHG include mucosa and submucosa capillaries and small veins, no obvious inflammation and no microthrombosis. The most common location for PHG is the fundus and body of the stomach [16]. Portal hypertension can promote short circuits in the gastric mucosal layer and submucosa, resulting in insufficient blood supply and oxygen to gastric mucosal cells. Portal hypertension can cause backflow obstruction and congestion of small blood vessels in the gastric mucosa, thus weakening the mucosal defence barrier [17]. Liver hypofunction activates kinins and then limits vasoconstriction, leading to gastric mucosa repair disorders. Renin–angiotensin–aldosterone activation aggravates liver fibrosis and then aggravates congestion of gastric mucosa.

A total of 325 patients with cirrhosis were included in the present study, and 284 patients were diagnosed with PHG. The incidence of PHG in cirrhosis was 87.4% and included 247 patients (76.0%) with mild PHG and 37 patients (11.4%) with severe PHG. Abbasi et al. [18] found that the incidence of PHG in cirrhosis was 79.3%. The incidence of PHG in cirrhosis fluctuates from 20.0% to 98.0% due to inconsistencies in description, population differences, lack of uniform diagnostic and grading criteria and interobserver and intraobserver differences.

Most studies have shown no significant relationship between PHG and gender. However, Simbrunner's team [5] pointed out that the male ratio of patients with severe PHG was significantly higher than that of patients with mild PHG or patients without PHG, and gender was an independent risk factor for severe PHG. Meanwhile, this study found that male patients with cirrhosis were more likely to develop PHG than female patients. Animal studies have shown that oestrogen and progesterone therapy can reduce portal pressure, gastric mucosal blood flow, vessel number and relative vessel area. Currently, there is insufficient evidence to support gender-related endocrine or haemodynamic influences on PHG, and the relationship between the severity of PHG and gender needs further prospective study.

In the present study, haemoglobin, platelet count, albumin, prothrombin time and ascites were closely related to the occurrence of PHG. And we found that albumin was predictor of PHG. Anaemia is a common complication in patients with cirrhosis. The main reason is gastrointestinal bleeding, followed by splenomegaly and hypersplenism, which also cause increased destruction of RBCs and platelets. Studies have shown that patients with severe PHG have significantly reduced haemoglobin, and with the aggravation of PHG, moderate to severe anaemia is more common. Prothrombin time and albumin can measure liver reserve function. When liver function is damaged in cirrhosis, albumin and coagulation factor synthesis are decreased. Min et al. [19] retrospectively studied 232 patients with chronic liver disease, and multivariate analysis indicated that albumin, platelet count and spleen volume were independent risk factors for PHG.

Some studies [10] have indicated that among 24 patients with severe PHG, 18 (75.0%) were Child–Pugh C and six (25.0%) were Child–Pugh B, indicating that severe PHG was more common in Child–Pugh C patients. PHG was associated with the Child–Pugh grade of liver function. Some studies have indicated [20] that the occurrence of PHG is related to staged liver function, and the incidence of PHG is high in patients with Child–Pugh B and C cirrhosis, while the occurrence of PHG has nothing to do with Child–Pugh A. In contrast, El-Kalla et al. [11] pointed out that the occurrence and severity of PHG were independent of liver function. Mezawa et al. [21] pointed out that PHG was relieved after TIPS, but liver function did not change significantly before or two weeks after surgery, so it was speculated that PHG had no significant relationship with liver function.

The present study indicated that Child–Pugh liver function was correlated with PHG occurrence. It is speculated that the deterioration of liver function aggravates portal hypertension, shorting the arteries and veins of gastric mucosa and submucosa, resulting in insufficient blood supply and oxygen supply to gastric mucosa cells. Portal hypertension is prone to blocked reflux and congestion of small blood vessels in gastric mucosa, both of which will aggravate gastric mucosa injury. The deterioration of liver function resulted in activation of the kinin system, imbalance of vasoconstriction and vasodilation and impaired repair of gastric mucosal cell proliferation. Impaired liver function activates the renin–angiotensin–aldosterone system, which exacerbates liver fibrosis, liver function and portal hypertension, eventually leading to a vicious cycle.

Many studies have explored the relationship between PHG and portal pressure and varices, but no consensus report has been reached. Kumar et al. [7] retrospectively analysed 254 patients with cirrhosis. The average varices grade in PHG patients was grade 3 and that in patients without PHG was grade 2 and the probability of PHG in large varices was 2.83 times higher than that in small varices. Saleem et al. [8] showed that the occurrence and severity of PHG were independent of the degree of EV. However, Tiwari et al. [2] found no relationship between PHG and oesophageal and gastric varices.

The present study showed that the occurrence and severity of PHG were related to the severity of EV and GV. The more serious the varices are, the higher the incidence of PHG and the more serious the PHG. We found that the degree of EV and GV were predictors of PHG. Portal hypertension is the initiating factor of PHG, and varicose veins are important clinical manifestations of portal hypertension. When portal venous pressure is higher than gastric venous pressure, gastric venous return is blocked, resulting in mucosal and submucosal vascular dilation and intravascular congestion. Portal hypertension causes gastric mucosa and submucosal portal hypertension causes the formation of submucosal arteriovenous anastomosis branches, reduces gastric mucosa perfusion and causes gastric mucosa ischaemia and hypoxia injury. This abnormality, in turn, may lead to epithelial cell damage and establish an environment for excessive production of oxygen free radicals, nitric oxide, tumor necrosis factor-alpha, endothelin-1, prostaglandins and/or other factors leading to cell damage [22].

Overall, albumin and the degree of EV and GV, which were independently associated with PHG, were predictors of PHG. When albumin is reduced or varicose veins are serious in liver cirrhosis patients, more attention should be given to the occurrence and severity of PHG.

The limitation of this study was that it was a single-centre, retrospective study. However, the incidence and risk factors for PHG were systematically analysed. Further prospective studies are needed to clarify the relationship between PHG and gender, liver function, and varices to provide help for clinical diagnosis and treatment.

## Conclusion

In this study, the incidence of portal hypertensive gastropathy with cirrhosis was 87.4%. The occurrence of PHG was related to gender, haemoglobin, platelet count, prothrombin time, albumin and Child–Pugh stage. The incidence and severity of PHG increased with the degree of EV, GV and ascites. The degree of albumin, EV and GV are independent risk factors for PHG.

## Data Availability

The datasets used and/or analysed during the current study available from the corresponding author on reasonable request.

## Abbreviations

WBC: White blood cells
RBC: Red blood cell
Hb: Haemoglobin
PLT: Platelet count
PT: Prothrombin time
TBIL: Total bilirubin
DBIL: Direct bilirubin
ALB: Albumin
ALT: Alanine aminotransferase
AST: Aspartate aminotransferase
Cr: Creatinine
EV: Oesophageal varices
PHG: Portal hypertensive gastropathy
GV: Gastric varices
